# Data on the treatment of used lubricating oil from two different sources using solvent extraction and adsorption

**DOI:** 10.1016/j.dib.2018.07.003

**Published:** 2018-07-09

**Authors:** Temitayo E. Oladimeji, Jacob A. Sonibare, James A. Omoleye, Abiola A. Adegbola, Hilary I. Okagbue

**Affiliations:** a Department of Chemical Engineering, Covenant University, Canaanland, Ota, Nigeria; b Department of Chemical Engineering, Obafemi Awolowo University, Ile-Ife, Osun State, Nigeria; c Department of Mathematics, Covenant University, Canaanland, Ota, Nigeria

**Keywords:** Used lubricating oil, Solvent extraction, Adsorption, Treatment, Sludge, Characterization

## Abstract

The data in this article were obtained from a research designed to investigate the effects of choice of solvent, mixing speed, temperature and solvent to oil ratio on the treatment process of used lubricating oils using solvent extraction and adsorption method. Various data on the performance of the three solvents chosen were studied and compared based on certain parameters are presented and discussed. From the results obtained, it was observed that MEK (Methyl Ethyl Ketone) had the best performance because it gave the highest sludge removal and closest properties to the fresh lubricating base stock. Furthermore, it was also determined that increase in temperature improved the quality of oil obtained up till 50 °C above this temperature poorer quality of oil was observed. But above all the factors investigated, it was concluded that solvent to oil ratio has a greater effect on the quality of oil produced after treatment.

**Specifications Table**

**Table:** 

Subject area	Chemical Engineering
More specific subject area	Membrane separation processes, environmental engineering
Type of data	Table and Figure
How data was acquired	The data was obtained using vacuum and atmospheric distillation setup
Data format	Analyzed
Experimental factors	Characterization of used lubricating oil.
Experimental features	The effects of choice of solvent, mixing speed, temperature and solvent to oil ratio on the treatment process of used lubricating oils
Data source location	Chemical Engineering Laboratory, Covenant University, Ota, Nigeria
Data accessibility	All the data are in this data article

**Value of the data**

• The data could be helpful in different brands of used lubricating oil characterization.
• The data presents varying capabilities of how the solvents used could effectively treat and remove contaminants from the used engine oil.
• Multivariate analysis can be used to establish relationships among the variables.
• The data presents evidence of cost effectiveness of the solvent extraction to adsorption method.

## 1 Data

The data describe research findings of the effects of choice of solvent, mixing speed, temperature and solvent to oil ratio on the treatment process of used oils using solvent extraction and adsorption method. The performance of the three solvents chosen were studied (process characterization) and compared based on certain parameters. To achieve this the oil was dehydrated using an atmospheric distillation setup and light ends were removed using a vacuum distillation setup. It was mixed with a solvent at different solvent to oil ratios and let to settle for 48 h before the solvent was recovered and activated charcoal was used to improve its colour amongst other properties. From results attained, it was observed that MEK (Methyl Ethyl Ketone) had the best performance because it gave the highest sludge removal and closest properties to the fresh lubricating base stock. Furthermore, it was also determined that increase in temperature, improved the quality of oil obtained up till 50 °C above this temperature poorer quality of oil was observed. But above all the factors investigated it was concluded that solvent to oil ratio has a greater effect on the quality of oil produced after treatment.

### 1.1 Detailed data description

Lubricants are used in many industries as they perform a variety of functions ranging from reducing friction between metal parts in contact with each other, heat dissipation, power transmission amongst many others [1], [2].

Lubricant Oil (LO) in use degrade over time, the degree of this degradation is dependent on the environment and operating conditions where the oil was used, however, a point is reached where the engine oil would no longer be able to perform its functions [3], [4]. Used LO is of a great importance as it represents a high percentage of volume of organic waste liquids generated in the world. It is a high pollutant material which has adverse effects on the environment [5] if not properly treated, handled, or disposed. United Nations (UN) 2016 [6] report estimated that about six million deaths were traceable to air pollution from indoor and outdoor sources. On the contrary, Used LOs can be considered as valuable resources as it can recover energy or profitable materials for further use [7]. Lubricating oil is rendered temporarily unsuitable to perform its functions mainly because of contaminants such as particles, oil, dirt, dust, carbon residue, metals, depleted additives and products from the incomplete combustion of fuels. Degradation causes alterations in the viscometric properties of the oil as a result of a transformation in the lubricating oils molecular structure caused by cracking, isomerization and polymerization reactions which are usually triggered by high temperatures. The consequence of this degradation is the formation of compounds with low molecular weight as well as oxidized products. Some of these contaminants (heavy metals, poly cyclic hydrocarbons, polyaromatic benzenes) constitute the noxious and carcinogenic effect of used lubricating oil [8]. The degradation of lubricating oil under working conditions arises principally because of the following reasons: oxidation and thermal decomposition of the lubricating oil at high temperatures, which changes the initial composition of the oil as well as generation of suspended particles [9].

Recycling of used lubricating oil deals with subjecting the oils to a series of processes that are able to eliminate most contaminants, including water, oxidation products, additives and thereby allowing the initial characteristics of the base oil to be re-established. Several methods have been proven feasible in the recycling of used lubricating oil, although not all of them are economically feasible principally because of high energy consumption during the recovery process.

Some of them include vacuum distillation, solvent extraction, acid-clay treatment, hydro finishing, to mention but few [10], [11], [12], [13], [14], [15]. Solvent extraction became very popular because it overcame the major problem that came along with acid treatment which is acid sludge generation and the solvent could be recovered using distillation for reuse [16]. Each of the different techniques that can be used to recycle the used lube oil will give different yields and qualities of the oil. Extraction processes using solvents involve the separation of paraffinic compounds and naphthenic compounds by their solubility differences from unwanted compounds such as resins and asphaltenes. The used lubricating oil is mixed with a solvent at ratios that will ensure that the highest possible solubilization of the base oil in the solvent is achieved.

## 2 Experimental design, materials and methods

### 2.1 Methodology

Two different used oil samples were used for the experimental analysis of this research. The first sample of oil was obtained from an auto repair shop in Lagos, Nigeria from a drum where oils from different vehicles had been drained. The second sample of oil was drained directly from one of the generators in Covenant University campus. The solvents used include MEK, propan-2-ol, a composite mixture of 75% MEK and 25% propan-2-ol and the adsorbent activated carbon.

### 2.2 Procedure

#### 2.2.1 Dehydration

A pre-measured quantity of the used oil is placed in a round bottom flask. A setup like the one below was erected in order to perform the dehydration process. The dehydration of both samples of used lubricating oil was carried out using a simple atmospheric distillation setup at a temperature of 120 °C because at this temperature water will evaporate. The dehydration process was done until no distillate was produced.

#### 2.2.2 Removal of light hydrocarbons and gasoline

**Fig. 1:**
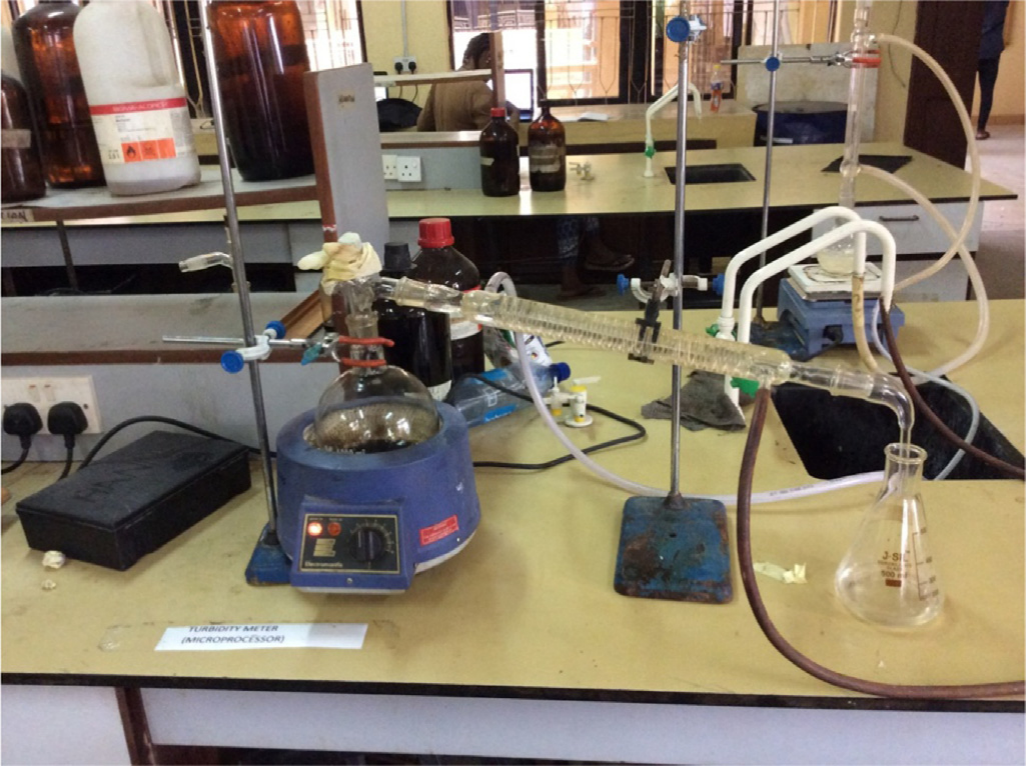
Atmospheric distillation of oil for dehydration.

Elimination of light ends as well as gasoline was made possible with the use of a simple batch vacuum distillation setup operated at a temperature of 210 °C and a pressure of about 3 inHg. The vacuum distillation process was carried out for an hour. The vacuum distillation setup is similar to that of the atmospheric distillation setup in Fig. 1 except with the addition of a vacuum pump.

#### 2.2.3 Solvent extraction

Three different types of solvents were used, two single solvents and a composite solvent. The single solvents used were MEK and propan-2-ol while the composite solvent comprised of 75% MEK and 25% propan-2-ol. The constituents of the composite solvent are stirred together for 30 min to ensure proper interaction between both of the solvents. A 100 ml sample of the oil is poured into a 600 ml beaker. Different quantities of either of the solvents were added to the oil sample in the beaker in these varying ratios, 1:1, 1:2, 1:3, 1:4 and 1:5. The oil and the solvent are then stirred for one hour to ensure that the mixture is adequately mixed using an electric hot plate at a speed of 500 rpm. The mixture was allowed to settle at a constant temperature for 48 h in a separating funnel. It separates into two phases, the extract and the raffinate phase. The extract phase is made up of the solvent and base oil components while the raffinate phase contains sludge. The raffinate phase was a black and semisolid and the extract phase was a brownish-black liquid. The sludge contains both solid and liquid contaminants found in the oil. The sludge collected from the separating funnel is then weighed and its volume recorded. The solvent and oil mixture (extract phase) undergoes further distillation for recovery.

#### 2.2.4 Solvent recovery using atmospheric distillation

The solvent is recovered from the extract phase using an atmospheric distillation setup. The oil and solvent from the extract phase is heated with an electric mantle to a temperature of 85 °C. This is slightly higher than the boiling point either of the solvents used so as to ensure the total removal of the solvent from the oil and solvent mixture.

#### 2.2.5 Adsorption

Activated carbon was the adsorbent of choice because of its strong affinity for PAH’s as well as its capacity to absorb fifty times its weight. A 40 g sample of oil was weighed alongside a five percent weight by weight of activated carbon. This was mixed for 15 min at ambient temperature at 500 rpm. The activated carbon is left in contact with the oil for four hours after which it must have settled by gravity and then the oil sample is collected.

### 2.3 Characterization

#### 2.3.1 Density

A volume of the oil sample was put into a graduated cylinder till it was three quarters full and a hydrometer is inserted at a relatively slow pace into this sample. While the device is floating, the point on the liquid surface where the hydrometer rests is the specific gravity.

$$Density\left(g/cm^{3}\right)=specific\;gravity\times 1g/cm^{3}$$

#### 2.3.2 Flash point

The oil sample was placed in an open cup where it was heated, its temperature was monitored using a thermometer. At specified time intervals the ignition source which is a flame is passed over it, this is done until the oil sample has flashed. An oil sample has flashed when a flame spreads on the entirety of the oil sample.

#### 2.3.3 Viscosity

If the temperature at which a viscosity reading is taken is not specified the reading is meaningless. Typically, the viscosity reading is usually reported at either 40 °C (100 °F) or 100 °C (212 °F). A capillary Viscometer is used to determine the kinematic viscosity of an oil sample used. The oil sample is placed inside a glass capillary U-tube and part of the sample is drawn through the tube using suction until it reaches the start position indicated on the tube’s side. The suction is then released, allowing the sample to flow back through the tube under gravity.

#### 2.3.4 Metal content

The metal content of different metals found in the oil samples was obtained by atomic absorption spectrometry using an atomic absorption spectrometer. A measured mass of used lube oil is heated to 60 °C and stirred. The used oil is then mixed with kerosene in the ratio 1:10. Sets of organometallic standards of metal (Cu, Fe, Pb) 4-cyclohexylbutyric acid salts were prepared and metal concentrations were determined by introducing the test solutions of engine oil samples into the flame of the atomic absorption spectrophotometer and thereafter recording the responses. Metal concentrations were determined from the calibration curve that is obtained from standard solutions. Standard solutions for all metals in engine oil samples were prepared according ASTM D 4628-2.

#### 2.3.5 Viscosity index

Viscosity index is a value that indicates the effect of temperature on the kinematic viscosity of an oil sample. This test is usually done using viscosity values attained at 40 and 100 °C. A table where viscosity values and reference series are listed is used in calculating the viscosity index value. The accuracy of this method is dependent on the accuracy of the kinematic viscosity value determination.

#### 2.3.6 Total base number

This test was carried out according to the ASTM D2896 method for testing the total base number of used and treated oils. The oil sample is dissolved in a chlorobenzene and glacial acetic acid. It is then titrated with Perchloric acid in glacial acetic acid.

The endpoint is obtained by potentiometric titration with a glass indicating electrode inside the solution and a reference electrode linked to the sample solution by a salt bridge.

### 2.4 Studied factors affecting the characteristics of the treated oil

Many factors were varied in order to study the effect that they would have on the solvent extraction and adsorption process as well as the characteristics of the treated oil. These factors include:

1. Choice of solvent
2. Mixing speed
3. Temperature
4. Effect of vacuum distillation.

## 3 Data analysis and presentation

The data are presented in subsections.

### 3.1 Choice of solvent

Several factors such as high molecular weight, solubility, sludge formation as well as ease of recovery were put into consideration before solvent choices were made. The ability of a polar solvent to form flocs from pre-treated lubricating oil depends on solubility parameters. According to Burrel’s classification of solvent alcohols are solvents with high capacity, ketones are solvents with medium capacity while hydrocarbons have relatively low capacity. To prove this theory, an alcohol propan-2-ol, a ketone methyl ethyl ketone and a 75% and 25% mixture of methyl ethyl ketone and propan-2-ol respectively. From the analysis, the greatest sludge formation can be attributed to MEK closely followed by the composite mixture then propan-2-ol. Upon the use of propan-2-ol it was discovered that compounds that have low molecular weight do not form a distinct layer of sludge after settling for up to four days, but on heating to recover the solvent a layer of sludge is deposited on the body of the containing vessel of the solvent and oil mixture.

### 3.2 Effect of solvent to oil ratio

**Fig. 2:**
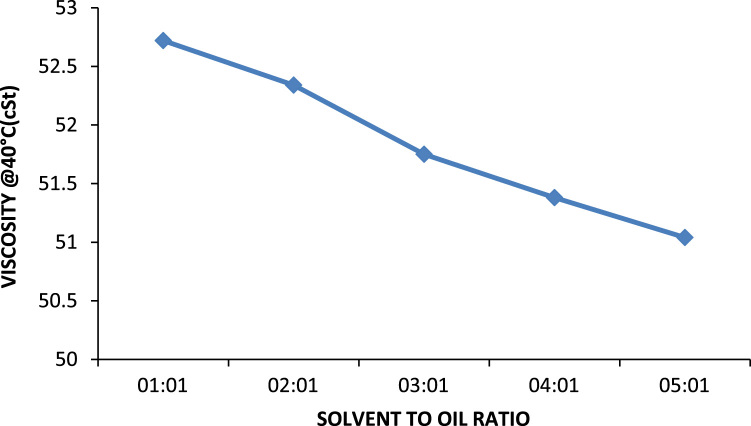
Effect of solvent to oil ratios on the viscosity of generator oil.

Solvent to oil ratio has a very prominent effect on the quality of oil produced (its characteristics) as well the percentage of oil recovered from a pre-treated oil sample. Usually the higher the solvent to oil ratio the greater its ability to remove contaminants in the form of sludge from the oil sample another important property gotten from increasing the solvent to oil ratio is the increase in the percentage recovery of the oil. For MEK the optimum solvent to oil ratio is 4:1 for all the solvents because this was the point where the properties of the oil changed best with respect to the quantity of solvent used. On comparing properties such as viscosity at 40 °C and 100 °C of used and treated oil samples there is a decreasing trend in the values obtained this shows that increasing the solvent to oil ratios increases the sludge forming capabilities. This is portrayed in Fig. 2 and can be seen that the viscosity of the oil decreases as solvent to oil ratio increases this is because the sludge removed from the treated oil contains aromatic compounds, aromatic compounds with high molecular weights have been removed, leaving behind paraffinic compounds of relatively lower viscosity when compared to its aromatic counterparts leading to an oil which has lower viscosities [12].

From observation the oil recovery was much higher with increasing solvent to oil ratio this is because at lower solvent to oil ratios the base oil might be saturated in the extraction phase leading to low oil recovery and greater oil losses [13]. It affects percentage oil losses because increasing solvent quantity for the same amount oil decreases oil losses.

**Fig. 3:**
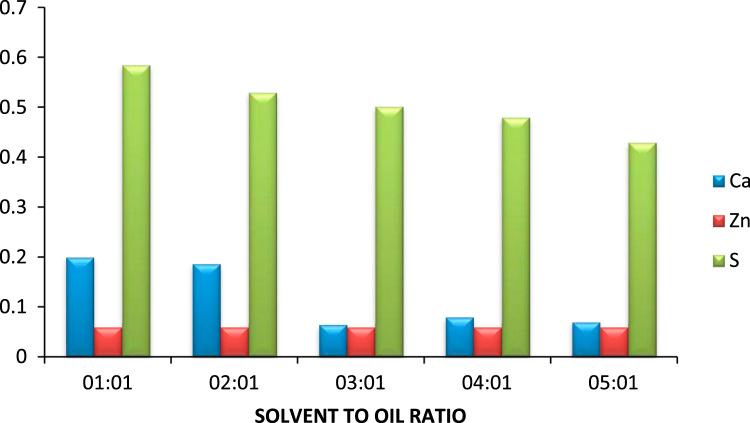
Effect of solvent to oil ratio on metals and non-metals on generator oil.

**Fig. 4:**
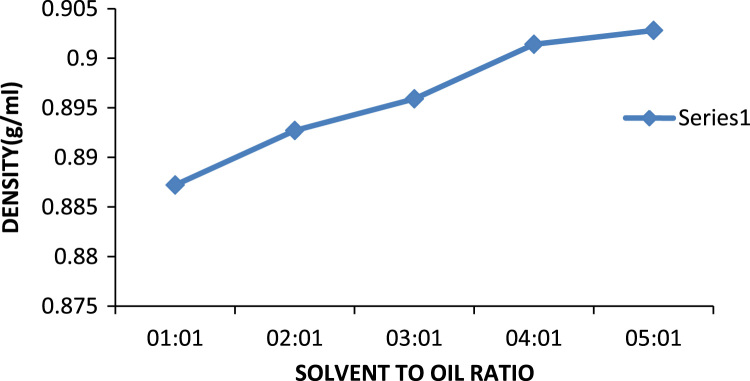
Density of generator oil at different solvent to oil ratios.

**Fig. 5:**
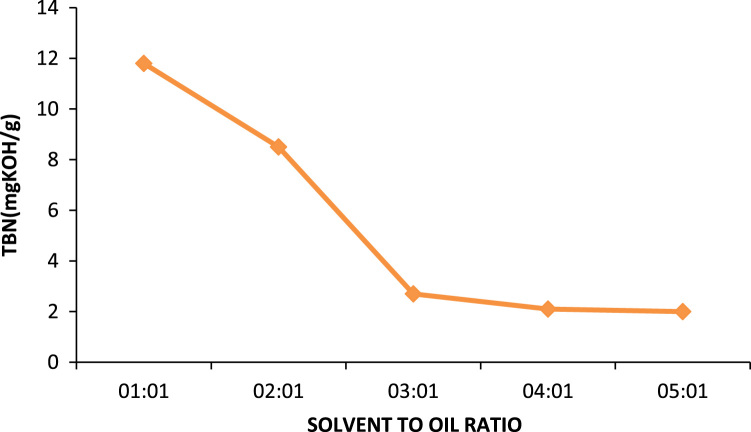
TBN of generator oil at different solvent to oil ratios.

The effect of solvent to oil ratio on the metals calcium and zinc and the non- metal sulphur was investigated as seen in Fig. 3. From the bar chart, it can be noticed the solvent MEK has the greatest effect on the metal calcium as compared to the non metal sulphur and little or no effect on zinc as such there is a prominent decrease in the calcium content of the oil. The source of the calcium in the oil is the detergent additive package which is used in most engine oils to suspend deposits so they can be filtered out of the oil this implies that as the solvent to oil ratio is increasing so also is the selective ability of the solvent to remove calcium from the oil. [14]. Zinc is also used in many additive packages. In addition, the density of generator oil at different solvent to oil ratios is shown in Fig. 4 while the TBN of generator oil at different solvent to oil ratios is presented in Fig. 5.

### 3.3 Effect of extraction temperature

The effect of temperatures ranging from 40 °C to 60 °C on the process was tested for the same solvent to oil ratio. At room temperature to 40 °C there is little effect felt by the process as a result of the temperature change, but above this the solvent started vaporizing faster as MEK is volatile. Temperature affects the solubility of base oil and spent oil impurities in solvents. The experiments were conducted in order to observe the characteristics of the treated oil above room temperature. Temperatures above 60 °C were not tested as they enhance the rate of solvent vaporization.

**Fig. 6:**
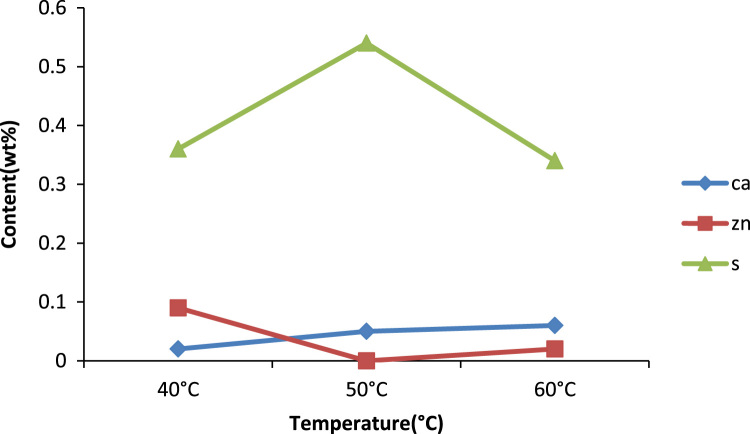
Temperature effect on the removal of metals and non metals.

**Fig. 7:**
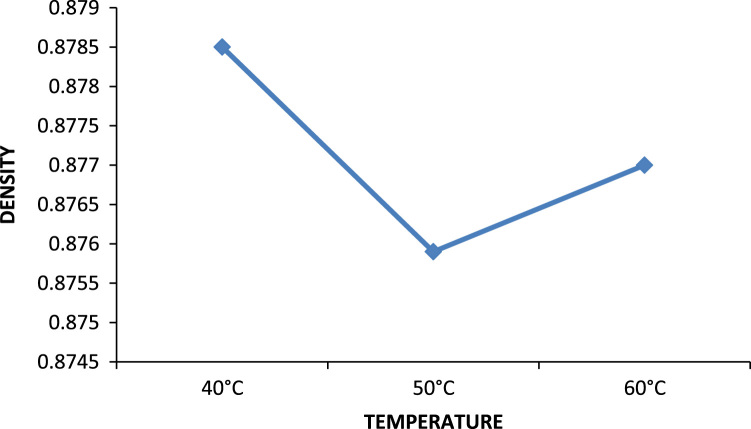
Temperature effect on density.

**Fig. 8:**
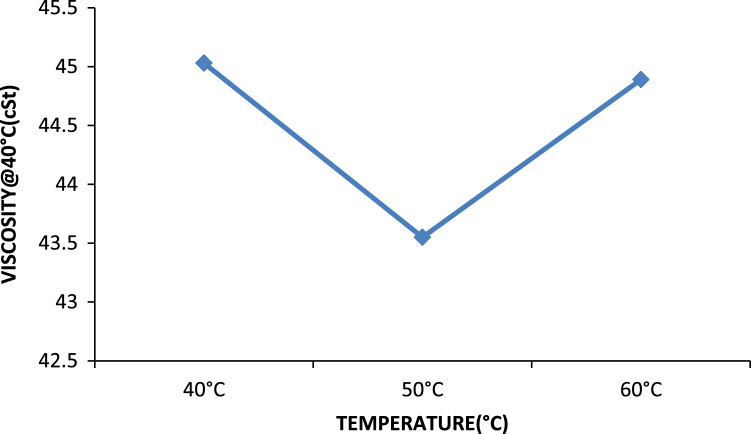
Effect of temperature on the viscosity of engine oil.

Previously conducted studies show that temperature has a great effect on the extraction process, especially those above 45 °C [1], [2], [3], [4] and reference therein. Lower quality oil is usually produced above this temperature. It can also be observed from studies that increase in temperature up to 50 °C reduces percent oil losses especially for MEK. Thus, most treatments using solvent extraction are best done at ambient temperature. In Fig. 6, the effect of temperature on the removal of metals and non metals were investigated, there is little change in the effects, an increase in temperature improved the removal of zinc but not calcium. In Fig. 7, the optimum point where the best density was gotten is at the 50 °C point, an increase in temperature favored reduction in density till this point, but thereafter above this temperature there is an increase in density [15]. Furthermore, Effect of temperature on the viscosity of engine oil is shown in Fig. 8.

### 3.4 Effect of mixing speed

**Fig. 9:**
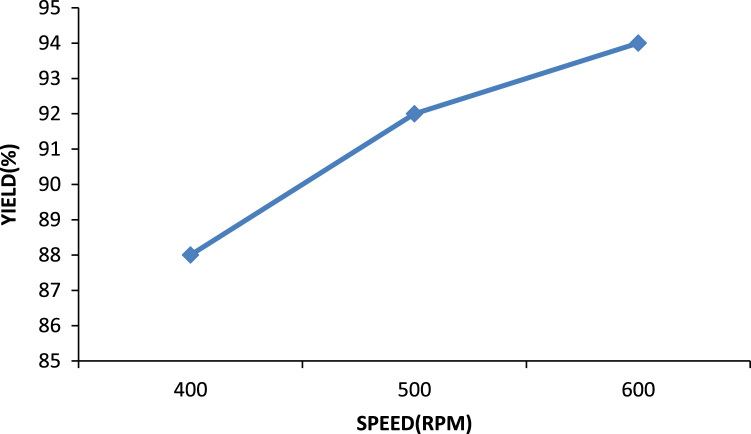
Yield of oil at various mixing speeds.

It was observed that for the same solvent to oil ratio there was an increase in the yield of the oil at different mixing speeds as well as an increase in the amount of sludge formed while mixing. This is presented in Fig. 9.

### 3.5 Influence of adsorption time

The optimum quantity and contact time between the adsorbent and the oil was investigated this is because the increase in the quantity of adsorbent used can retard the rate of the adsorption process. This is in line with studies made by previous researchers. It was determined that a 5%w/w oil of adsorbent was used for a contact period of four.

### 3.6 Comparison of characteristics between the different solvents

#### 3.6.1 Metal content and non-metal content

**Fig. 10:**
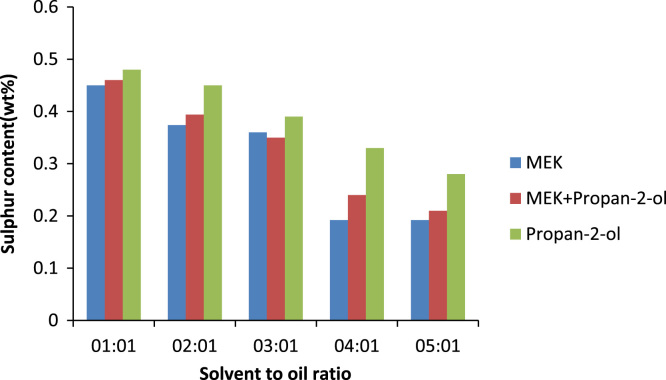
Effect of solvent to oil ratio on sulphur content.

**Fig. 11:**
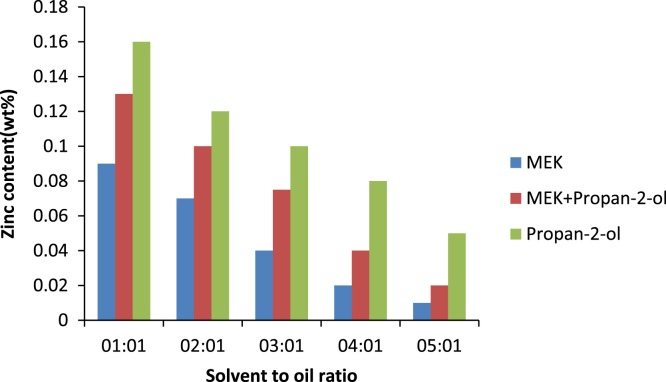
Effect of solvent to oil ratio on zinc content.

**Fig. 12:**
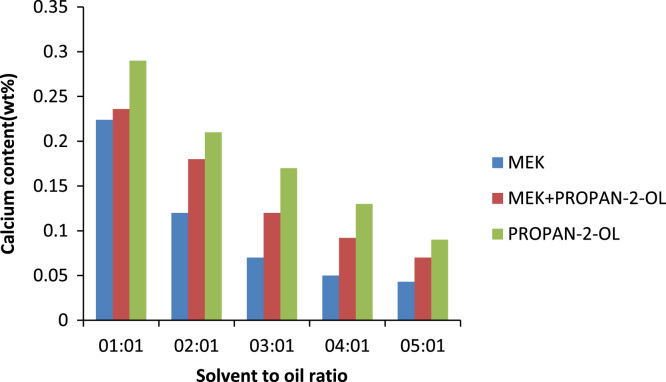
Effect of solvent to oil ratio on calcium content.

The metal content of base oil is a very important parameter as the metal content in an oil sample as it can increase the rate of corrosion of the metal parts it is in contact with. The metal and nonmetal content of oil mainly come from the additives added to the oil. Calcium comes mainly from detergents and dispersants, sulphur from extreme pressure additives and Zinc is introduced to base oil in the form of additives packaged as anti-oxidant, corrosion inhibitor, anti-wear, detergent. MEK has the most effect on the metals, zinc and calcium as well as the non-metal sulphur as can be seen in Fig. 10, Fig. 11, Fig. 12.

#### 3.6.2 Viscosity

**Fig. 13:**
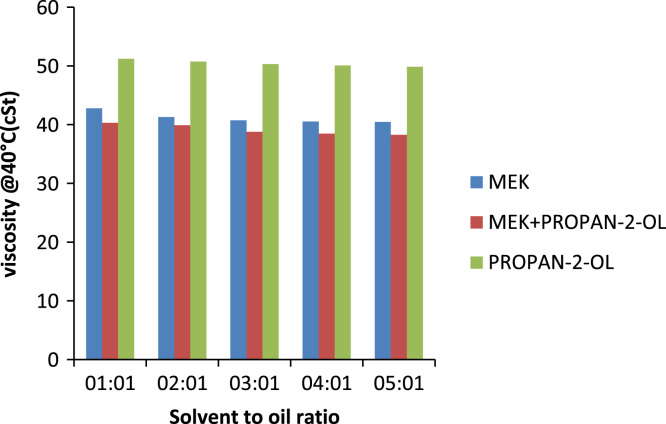
Effect of solvent to oil ratio on the viscosity of automobile oil at 40 °C.

**Fig. 14:**
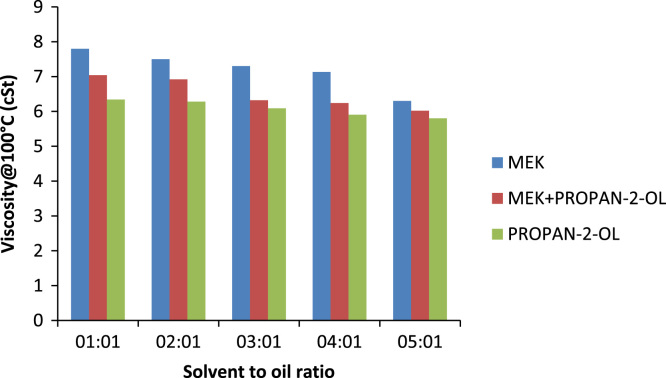
Effect of solvent to oil ratio on the viscosity of automobile oil at 100 °C.

The type of viscosity measured is the kinematic viscosity. It is the ratio of dynamic viscosity to density. In addition, it is affected by pressure and temperature. Like the general definition of viscosity as temperature increases, viscosity decreases and vice-versa the same also applies to kinematic viscosity. Generally in the treatment process vacuum distillation has the most effect on the viscosity of the oil, but the effect of solvent extraction alone on certain properties such as viscosity and flash point was investigated. At the end of the treatment with the various solvents, it was determined that no matter the solvent used the effect it would have on the viscosity is marginal compared to the viscosity of an unused lube base stock. But the effect of the different solvents on oil can be seen in Figs. 13 and 14 of which MEK has the greatest effect on the oil, closely followed by the composite mixture of MEK and propan-2-ol , propan-2-ol. Therefore, vacuum distillation is a step that should not be skipped as it has a large effect on the physicochemical properties of oil [16].

#### 3.6.3 TBN

**Fig. 15:**
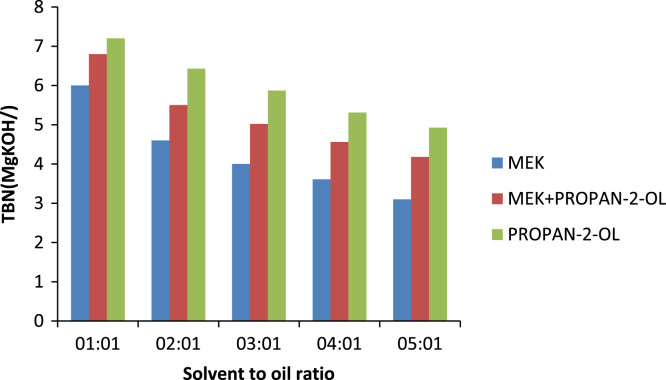
Effect of solvent to oil on TBN.

Combustion engines of most automotive vehicles are blended with additives that are highly alkaline in nature this is to ensure that the oil would be able to neutralize the corrosive products formed from oxidation. The higher the TBN value of the oil the better it would be at neutralizing acids, but since the oil has been used the TBN value is relatively low but despite this these highly alkaline additives are not completely depleted. Fig. 15 shows the effects of the different solvents in the removal of the highly alkaline additives. From the graph, it can be noticed that MEK has a greater effect on the reduction of the TBN value between 5:1 and 1:1 there was a reduction of 2.9 mgKOH/g while within this range for propan-2-ol there was a 2.274 mgKOH/g change and the composite solvent had a 2.62 mgKOH/g therefore it is implied that MEK has the greatest ability to reduce the value of TBN, closely followed by the composite solvent and propan-2-ol.

The data presented in this article has shown that all the solvents used could effectively treat and remove contaminants from the used engine oil though not with the same capabilities. From the characteristics compared, it can be seen that MEK gave the most favourable characteristics at an optimum solvent to oil ratio of 4:1. Different factors affect the quality of treated oil gotten from used lubricating oil. Temperature helps improve the solubility of oil in the solvent up till a temperature of 60 °C with an improvement in the properties above this temperature poorer quality of oil is attained. Also the mixing speed increases the yield at the same solvent to oil ratio. Solvent to oil ratio had a greater effect on the properties of the oil than temperature. As a result of the cost effectiveness of the solvent extraction to adsorption method, it can be prescribed for use. Furthermore, several statistical methods can be applied to improve the usefulness, variable characterization and reliability of the data. Data obtained from various experiments are refined, analyzed and interpreted with the aid of statistical methods. Readers are refer to the following articles [17], [18], [19], [20], [21], [22], [23], [24], [25], [26], [27], [28], [29], [30], [31], [32], [33], [34], [35], [36].
